# Comparison of the Efficacy of Throat Pack in Uncuffed Tube Versus Microcuff® Tubes on Sealing Pressure in Pediatric Laparoscopic Surgeries: A Randomized Clinical Trial

**DOI:** 10.7759/cureus.84376

**Published:** 2025-05-18

**Authors:** Roushan Patel, Rishi Anand, Deb Sanjay Nag, Biswajit Sen, Roshan Lal Gope, Tapas Barman

**Affiliations:** 1 Anesthesiology, Tata Main Hospital, Jamshedpur, IND; 2 Anesthesiology, Manipal Tata Medical College, Jamshedpur, IND

**Keywords:** anesthesia, artificial endotracheal intubation throat pack airway management, cuffed, endotracheal, endotracheal tubes, general, laparoscopy, pediatric surgery, tube, ventilation

## Abstract

Background and aims

Pneumoperitoneum and extreme Trendelenburg position in laparoscopic surgeries require ventilation at higher peak pressure, potentially resulting in peritubular leak and difficult ventilation. Our study aimed to assess whether a throat pack can provide an adequate seal to prevent peritubular leak around uncuffed tube pediatric laparoscopic surgeries as compared to a Microcuff® tube.

Methods

This randomized clinical trial was carried out on 94 children aged between eight months and five years undergoing laparoscopic surgery under general anesthesia allocated in two parallel groups using a computer-generated random number. We compared sealing pressure, peritubular leak, adequacy of ventilation, quality of capnography, and post-extubation laryngospasm or stridor.

Results

The uncuffed tube with a throat pack effectively seals the airway, and neither the creation of pneumoperitoneum nor the Trendelenburg position affected ventilation or the seal's effectiveness. There was no significant difference in the incidence of postoperative stridor between the two groups.

Conclusion

The throat pack provides an effective seal in an uncuffed tube to perform laparoscopic surgery without increasing airway morbidity.

## Introduction

Pediatric laparoscopic surgeries pose unique challenges due to the physiological impact of pneumoperitoneum and Trendelenburg position, which significantly decrease total lung compliance necessitating ventilation at higher pressure [1], potentially leading to peritubular leak. To achieve effective ventilation, we are compelled to use a cuffed tube. Cuffed tubes are not without limitations. The cuffed tube has the potential to cause tracheal injury [2]. In addition, the shorter length of the trachea in children reduces the margin of safety by 50% when using a cuffed tube as compared to an uncuffed tube [3]. Uncuffed tubes have disadvantages like peritubular leak, ineffective ventilation, theatre contamination, and increased aspiration risk [4]. A throat pack with wet ribbon gauze may mitigate this issue with an uncuffed tube to a large extent. The Cochrane review in 2017 suggested that the evidence quality was low due to study design issues, warranting cautious interpretation of cuffed versus uncuffed tubes, with further well-conducted trials needed to address gaps in understanding their safety and efficacy in children [4].

Microcuff® tubes, although effective, are significantly more expensive and not readily available in many settings. This prompted the evaluation of alternative methods, such as the use of throat packs to achieve adequate seals and prevent leaks around uncuffed tubes in pediatric laparoscopic surgery. The purpose of this study was to determine whether a throat pack provides an adequate pressure seal to prevent leaks around uncuffed while meeting increased ventilation demands and accommodating reduced thoraco-pulmonary compliance associated with pediatric laparoscopic surgery.

## Materials and methods

This randomized clinical trial was conducted with permission from the Institute Ethics Committee of the Jawaharlal Institute of Postgraduate Medical Education and Research (JIPMER) (approval number: JIP/IEC/2016/27/918) and written consent from the parents of children aged eight months to five years who were having laparoscopic surgery with general anesthesia at JIPMER from July 2016 to November 2017. The trial was registered with the Clinical Trials Registry-India (trial registration number: CTRI/2017/12/010858). All children were admitted to the hospital one day before the surgery. The study selected patients with physical statuses 1 and 2 according to the American Society of Anesthesiologists (ASA). After taking written informed consent from the legally acceptable guardians, the patients were allocated into either of two groups, i.e., the Microcuff® group (Group M) or the uncuffed group with a throat pack (Group U). Randomization was done using a computer-generated random number table. The sample size was estimated using the statistical formula for the comparison of two independent means. The minimum expected mean difference in sealing pressure between the groups is 3 with a standard deviation of 4.5. The sample size is estimated at a 5% level of significance with 90% power.

In the operating room, standard monitors such as pulse oximetry, electrocardiogram, and non-invasive blood pressure were attached, and baseline recordings were obtained. An established anesthetic protocol was implemented. In cases where there was no prior intravascular access, anesthesia was initiated using sevoflurane (4-8%) in 100% oxygen. Tracheal intubation was supported by administering fentanyl at a dose of 2 mcg/kg and atracurium at 0.5 mg/kg. Conversely, when intravascular access was established before induction, fentanyl (2 mcg/kg) and thiopentone (5 mg/kg) were used for anesthesia induction, followed by paralysis with atracurium at 0.5 mg/kg. In the Microcuff® group, tracheal intubation was carried out using an oral Microcuff® pediatric endotracheal tube. The inner diameter (ID) of the tube was determined based on the Motoyama formula (ID=age/4+3.5) [5]. The tracheal tube was positioned so that its vocal cord marker on the endotracheal tube was aligned with the level of the vocal cords. 

Uncuffed endotracheal tube sizes were determined using the modified Cole's formula (ID (mm)=(age in years/4)+4.0) [6]. Tube insertion depths were initially at insertion depth (cm)=3×tube size (mm) as a standard protocol. Bilateral breath sounds were assessed to ensure proper lung function. For both groups, air leak pressure after intubation and confirmation of tube position were evaluated with the patient in a supine position and the head in a neutral alignment. To assess the air leak, an air leak test (ALT) was done by assessing the pressure required to produce an audible leak of air when the endotracheal tube cuff is inflated and auscultated with a stethoscope. In cases where no air leak was found at an inflation pressure of 20 cm H₂O, the tube was regarded as too large and was substituted with the next smaller size. Conversely, if the tracheal tube demonstrated a significant air leak that compromised proper ventilation, it was replaced with the next larger size.

After confirming that neither group contained oversized tubes, the integrity of the seal was evaluated using mechanical ventilation. For the cuffed endotracheal tubes, we inflated them using a cuff pressure manometer (Portex) and made sure the pressure stayed below 20 cm H₂O by using a pressure release valve. The minimal sealing pressure was determined during steady-state ventilation and consistently maintained. This process involved gradually decreasing the cuff pressure until an audible leak was detected at the patient's mouth, followed by a gradual increase in pressure until the leak ceased. All measurements of the minimal cuff pressure needed for effective airway sealing, along with the quality of the seal, were carefully documented.

For uncuffed tubes, after confirming the appropriate size, the tube was adequately packed with wet ribbon gauze as a throat pack using Magill's forceps under laryngoscopy view.

Oropharyngeal leak pressure is measured in both the Microcuff® group (after the cuff is properly inflated) and the uncuffed group (after packing the throat) when the patient is switched to bag mode and the adjustable pressure-limiting valve is completely closed with a flow of 3 L/min, noting the specific pressure (sealing pressure) at which a leak can be heard (checked by listening over the front of the neck) [7,8]. We maintained anesthesia with a 50:50 mixture of oxygen (O2) and air and sevoflurane, using a circle absorber with a flow rate of 1 L/min. All children were ventilated with a 6-10 ml/kg tidal volume and with a positive end-expiratory pressure (PEEP) of 5 cm H₂O. Respiratory rates were adjusted by the anesthetist to maintain an end-tidal carbon dioxide (EtCO₂) of 30-40 cm. Atracurium was administered in bolus doses intermittently to facilitate muscle relaxation. Patients were extubated following the reversal of the medication.

Rescue measure

If the throat pack in the uncuffed tube failed to provide a sealing pressure of more than 25 cm H₂O, the patient would have been intubated with a Microcuff® tube of appropriate size and reported as a failure.

Twenty-four hours postoperatively, the children were assessed for change in voice, cough, and hoarse cry to determine the incidence of postoperative sore throat.

Aim and objectives

The primary objective is to assess and compare the efficacy of throat packs in uncuffed tubes on sealing pressure during laparoscopic surgeries in pediatric patients. The secondary objective is to report associated airway morbidity in both groups. 

## Results

**Figure 1:**
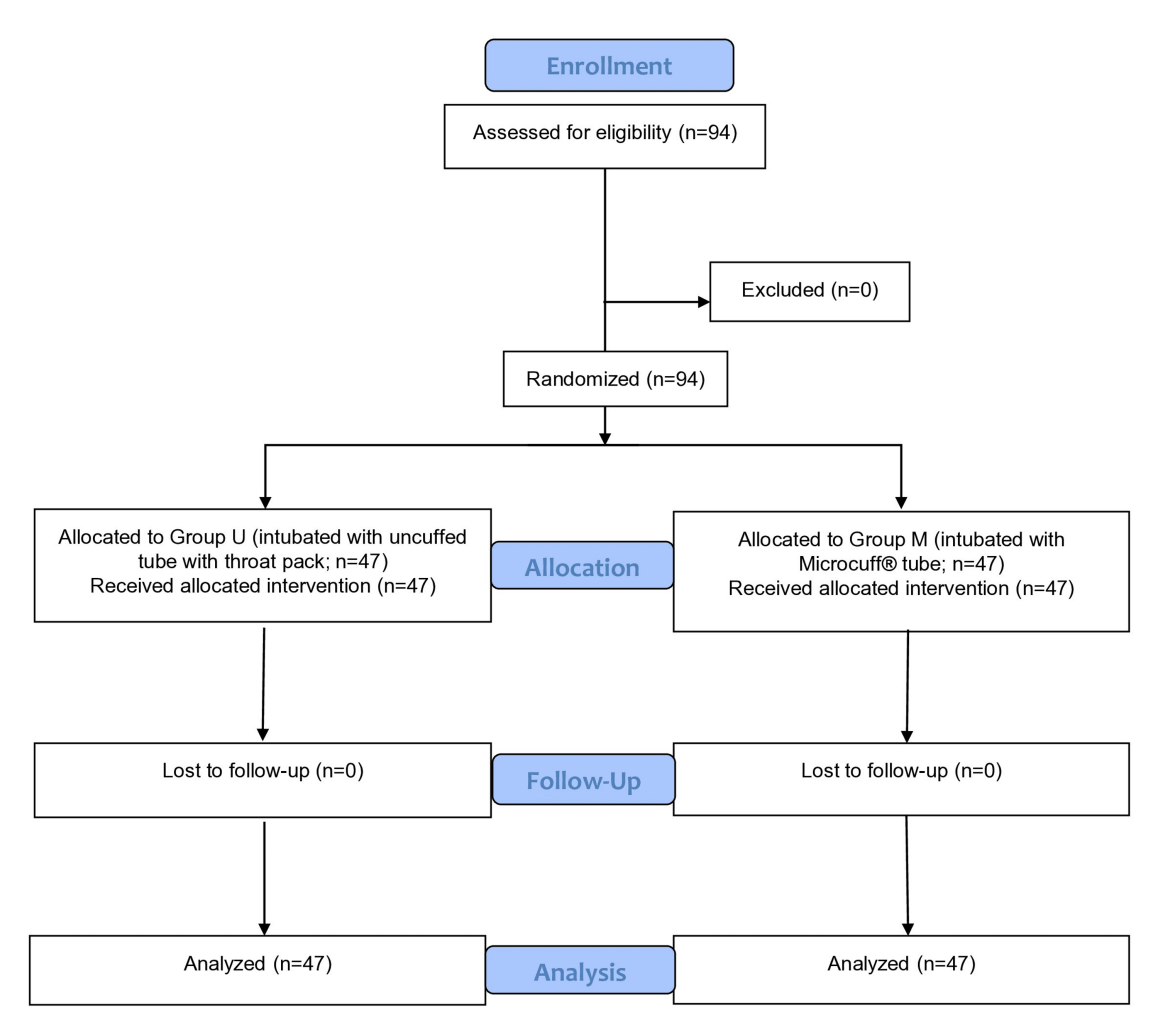
CONSORT flow diagram CONSORT: Consolidated Standards of Reporting Trials

The flowchart for the Consolidated Standards of Reporting Trials (CONSORT) statement is attached as Figure 1. 

All the patient data was entered in an Excel sheet and proofread for typographical errors and mistakes. Examination of the descriptive characteristics and outcomes of the patients was conducted by the endotracheal tube group, i.e., the Microcuff® tube and throat pack group. Appropriate parametric and non-parametric tests were used to assess differences between the two groups. For continuous variables, Student's t-test was used when the distribution was normal, while the Wilcoxon rank test was used in case the distribution deviated from normal. Similarly, chi-squared tests and exact tests were used for categorical variables; the exact test was used when one or more of the table cells had fewer than five patients. Baseline demographics and surgical characteristics were comparable in Groups M and U. The data are represented in Table 1.

**Table 1:** Demographic parameters Data expressed as mean±SD Group M: Microcuff® group; Group U: uncuffed group with a throat pack

Measure	Group M (N=47)	Group U (N=47)	t-value	P-value	Note
Age (months)	36.3±17.7	32.4±20.0	0.799	0.33	Not significant
Weight (kg)	12.1±2.9	11.4±2.9	0.933	0.24	Not significant
Duration of surgery (min)	93.8±50.0	123.9±45.6	-2.44	0.16	Not significant

The baseline ventilation parameters, like end-tidal CO₂, peak airway pressure, and leak airway pressure, were comparable between Group M and Group U. This is represented in Table 2.

**Table 2:** Baseline ventilation parameters Data expressed as mean±SD Group M: Microcuff® group; Group U: uncuffed group with a throat pack; EtCO₂: end-tidal CO₂ in capnography; PAW: peak airway pressure

Measure	Group M (N=47)	Group U (N=47)	t-value	P-value	Note
Expired tidal volume (ml)	111.7±24.7	103.9±26.3	0.896	0.15	Not significant
EtCO₂ (mm Hg)	33.4±2.4	33.4±2.4	0	0.93	Not significant
PAW (cm H₂O)	15.5±2.5	15.5±1.8	0	0.92	Not significant
Leak pressure (cm H₂O)	27.0±2.2	27.3±2.0	-0.455	0.6	Not significant

The leak pressure for Group M was 27.03±2.22 cm of water, while Group U had a leak pressure of 27.26±1.95 cm of water. These results show no statistically significant difference between the two groups. After pneumoperitoneum, the peak airway pressures were similar in both groups, with the Microcuff® group measuring 19.7 cm of water and the uncuffed group at 18.8 cm of water. There was an increase in measurements within each group before and after pneumoperitoneum and after the head-down position.

The ventilation parameters after pneumoperitoneum and after the Trendelenburg position are depicted in Table 3 and Table 4. 

**Table 3:** Ventilation parameter after pneumoperitoneum Data expressed as mean±SD Group M: Microcuff® group; Group U: uncuffed group with a throat pack; EtCO₂: end-tidal CO₂ in capnography; PAW: peak airway pressure

Measure	Group M (N=47)	Group U (N=47)	t-value	P-value	Note
Expired tidal volume (ml)	111.1±25.3	102.5±26.2	0.396	0.1	Not significant
EtCO₂ (mm Hg)	34.6±2.7	34.7±1.8	-0.167	0.9	Not significant
PAW (cm H₂O)	19.7±3.3	18.8±1.9	0.397	0.4	Not significant
Leak pressure (cm H₂O)	27.1±2.1	27.4±2.0	-0.447	0.5	Not significant

**Table 4:** Ventilation parameter after Trendelenburg Data expressed as mean±SD Group M: Microcuff® group; Group U: uncuffed group with a throat pack; EtCO₂: end-tidal CO₂ in capnography; PAW: peak airway pressure

Measure	Group M (N=47)	Group U (N=47)	t-value	P-value	Note
Expired tidal volume (ml)	108.0±24.8	99.9±24.6	0.41	0.1	Not significant
EtCO₂ (mm Hg)	34.6±2.3	35.0±1.9	-0.405	0.3	Not significant
PAW (cm H₂O)	20.1±2.5	21.6±1.4	-0.79	0.4	Not significant
Leak pressure (cm H₂O)	27.3±2.0	27.7±1.9	-0.455	0.4	Not significant

The efficacy of sealing, defined by (definition with reference) when a throat pack was used with an uncuffed tube, was comparable to that of the Microcuff® group at baseline. The effect was found to be similar after pneumoperitoneum and after Trendelenburg (Table 5).

**Table 5:** Efficacy of sealing Data expressed as mean±SD Group M: Microcuff® group; Group U: uncuffed group with a throat pack

Measure	Group M (N=47)	Group U (N=47)	t-value	P-value	Note
Baseline leak pressure (cm H₂O)	11.3±3.2	11.8±2.3	-0.398	0.4	Not significant
After pneumoperitoneum leak (cm H₂O)	7.6±3.1	8.6±2.3	-0.662	0.1	Not significant
After Trendelenburg leak (cm H₂O)	7.2±3.0	8.0±2.3	-0.668	0.2	Not significant

The three parameters, namely, leak pressure, peak airway pressure, and the difference between the two (leak pressure-peak airway pressure) demonstrating the efficacy of sealing, were measured at baseline, after pneumoperitoneum, and after Trendelenburg. 

## Discussion

Establishing pneumoperitoneum and placing the patient in the Trendelenburg position during laparoscopy can decrease pulmonary compliance. This reduction may result in inadequate ventilation or an increase in air leaks around the tracheal tube if an uncuffed tube is used. This is supported by previous research indicating that the unique physiological changes associated with laparoscopic procedures can challenge conventional airway management techniques, particularly in pediatric patients [9,10]. In our study, we found that a throat pack in an uncuffed tube adequately sealed the airway and the creation of pneumoperitoneum and Trendelenburg position did not adversely affect ventilation or the effectiveness of the seal. For our study, effective ventilation was defined as a square wave capnogram, with EtCO₂ values maintained within the clinically acceptable range of 35-45 mm Hg and the ability to ventilate with low flow (<2 L/min), which is consistent with findings from Bradford et al. [11] demonstrating that appropriate airway sealing techniques can enhance ventilatory efficiency.

While we noted an increase in peak airway pressure due to pneumoperitoneum, it remained well below the sealing pressure in both conditions, confirming the importance of effective airway management during laparoscopic surgery. After pneumoperitoneum, peak airway pressures in both groups were indeed higher than baseline values, indicating that pneumoperitoneum typically leads to reduced pulmonary compliance and increased airway resistance. However, it is reassuring that the work of breathing remained manageable, as evidenced by the comparable levels of leak during ventilation (LAW) between the two groups, both before and after pneumoperitoneum. Moreover, our assessment of EtCO₂ was optimal in both groups before and after pneumoperitoneum, indicating adequate ventilation with throat packing in uncuffed tubes.

It is worth noting that tube exchange was necessary for four patients, leading to their exclusion from the study. The overall incidence of post-extubation stridor in pediatric patients is reported to be about 1-30.3% in the literature [12]. Our findings indicated no significant difference in the incidence of postoperative stridor between the Microcuff® tube and the uncuffed tube, suggesting that the use of a throat pack doesn't increase the risk of airway complications. This aligns with recent findings by Veder et al. [13], which suggest that uncuffed tubes, when used with adequate sealing techniques, do not increase the risk of stridor, thus challenging the traditional preference for cuffed tubes in similar patient populations.

In other studies, comparing the efficacy of uncuffed tubes with throat packs and Microcuff® tubes in pediatric laparoscopic surgeries, Microcuff® tubes have been shown to provide better airway sealing and effective ventilation at lower cuff pressures compared to conventional cuffed tubes [14]. They allow for the safe use of low-flow anesthesia and maintain plateau-type capnography [15]. When using the sealing pressure technique, Microcuff® tubes demonstrate lower post-extubation morbidity and more reliable air leak prevention compared to finger palpation or fixed pressure techniques [16]. However, uncuffed tubes can also be safely used in both laparoscopic and laparotomy surgeries, with no significant differences in tube changes or side effects when compared to cuffed tubes [17]. The choice between Microcuff® and uncuffed tubes may depend on specific surgical requirements and patient characteristics.

Traditionally, it is believed that in children under eight years old, the narrowest part of the airway is at the cricoid cartilage, which is rigid and circular. However, as children grow, their airways develop into a more cylindrical shape, with the narrowest section shifting to the level of the vocal cords [18]. In view of this, the challenge remains that some authors argue that stridor is insufficient for identifying tracheal mucosal injuries and advocate for evaluating all airway complications through endoscopy. While they acknowledge that symptoms of injury may not appear right away, it remains uncertain whether their recommendation for endoscopy for all children post-extubation remains unclear [18].

Weaknesses of the study include the following: small sample size, single-center trial, no blinding due to the nature of the study itself, and randomization of the confounding factors.

## Conclusions

This randomized clinical trial provides evidence supporting the use of throat packs with uncuffed endotracheal tubes as a viable strategy for securing the airway during pediatric laparoscopic surgeries. The study demonstrates that this approach effectively addresses the unique challenges posed by pneumoperitoneum and the Trendelenburg position, which can compromise pulmonary compliance and ventilation. The researchers observed that using a throat pack along with an uncuffed tube effectively kept the airway sealed, ensured good ventilation, and did not raise the chances of problems like stridor after surgery, unlike Microcuff® tubes. This is particularly significant as cuffed tubes, while often used to achieve better sealing, carry their risks, such as tracheal injury, especially in children.

Moreover, the study highlights the potential benefits of this technique, including cost-effectiveness and easy accessibility, which can broaden its applicability, particularly in resource-limited settings. By demonstrating that throat packs can provide comparable sealing without increasing airway morbidity, this research challenges the conventional preference for cuffed tubes in pediatric laparoscopic procedures. The results suggest that incorporating throat packs with uncuffed tubes can provide a practical, safe, and efficient alternative for airway management in pediatric patients undergoing laparoscopic surgery, where the availability of Microcuff® tubes could be a concern, especially in developing countries. The findings warrant consideration by healthcare providers when making decisions regarding airway management strategies in this patient population.
